# Human Endogenous Retrovirus K in Astrocytes Is Altered in Parkinson's Disease

**DOI:** 10.1002/mds.30128

**Published:** 2025-01-22

**Authors:** YuHong Fu, Gabrielle L. Adler, Priscilla Youssef, Katherine Phan, Glenda M. Halliday, Nicolas Dzamko, Woojin Scott Kim

**Affiliations:** ^1^ Brain and Mind Centre The University of Sydney Sydney New South Wales Australia; ^2^ School of Medical Sciences The University of Sydney Sydney New South Wales Australia

**Keywords:** Parkinson's disease, human endogenous retrovirus, HERV‐K, envelope, astrocyte, GFAP, biomarkers

## Abstract

**Background:**

Parkinson's disease (PD) is the most common neurodegenerative movement disease. Human endogenous retroviruses (HERVs) are proviral remnants of ancient retroviral infection of germ cells that now constitute about 8% of the human genome. Under certain disease conditions, HERV genes are activated and partake in the disease process. However, virtually nothing is known about the pathological relationship, if any, between HERV and PD.

**Objective:**

The objectives of this study were to unravel the pathological relationship between human endogenous retrovirus K (HERV‐K) and PD, determine the localization of HERV‐K in the brain, determine whether HERV‐K levels are altered in PD brain and blood, and examine whether HERV‐K could serve as a biomarker for PD.

**Methods:**

In situ HERV‐K and glial fibrillary acidic protein (GFAP) expression in the superior frontal and fusiform cortices of PD and control brain were analyzed using immunofluorescence and confocal microscopy. HERV‐K load and copy number in PD and control blood were measured by digital droplet polymerase chain reaction and GFAP by single‐molecule array. HERV‐K load was analyzed in relation to the Hoehn and Yahr Scale and Movement Disorder Society Unified Parkinson's Disease Rating Scale Part III.

**Results:**

HERV‐K is predominantly expressed in astrocytes and colocalized with astrocytic GFAP, with decreased expression of both HERV‐K and GFAP in PD brain compared with controls. Consistent with this, HERV‐K levels were decreased in PD blood compared with controls and were correlated to blood GFAP levels. HERV‐K levels were inversely correlated to PD severity and duration.

**Conclusions:**

These findings suggest that HERV‐K is related to astrocyte function and to PD progression, and that HERV‐K could be neuroprotective. © 2025 The Author(s). *Movement Disorders* published by Wiley Periodicals LLC on behalf of International Parkinson and Movement Disorder Society.

Parkinson's disease (PD) is the most common neurodegenerative movement disease with a global prevalence of approximately 8.5 million cases. PD is clinically characterized by loss of dopaminergic neurons, causing motor symptoms of rest tremor, rigidity, and bradykinesia. It is pathologically characterized by the presence of α‐synuclein aggregates in neurons. PD is a complex polygenic disease, with more than 20 genes implicated in the disease process. Although our understanding of the genetic basis of PD has advanced considerably, the molecular mechanisms underlying sporadic PD remain unresolved. One non‐Mendelian mechanism that is understudied or unknown in PD is the interplay of human endogenous retroviruses (HERVs).

HERVs belong to the *Retroviridae* family that infiltrated the human genome millions of years ago.^1^, ^2^ Once inside cells, the reverse transcriptase *pol* gene transcribes the viral RNA genome into DNA, which then integrates into the human genome at numerous sites. The integrated proviral DNAs are now part and parcel of the normal human genome that constitute about 8% of the human genome. Data on the expression patterns of HERV in healthy tissues and cell types are beginning to emerge that are helping to define the function of HERV in humans.^3^ The importance of HERV in human physiology is underscored by the fact that HERV genes are expressed, variably, in all the tissues examined,^4^ with the brain being the highest.^3^ The fact that the human genome has conserved HERV open reading frames and functional domains over millions of years may explain beneficial functions that HERVs provide to human hosts.

Understanding of the function of HERVs in normal human physiology is becoming clearer, and it is apparent that HERVs participate in a number of important physiological processes. A well‐described example is the role of the HERV env protein (also known as syncytin) in the formation of syncytiotrophoblast in embryonic development.^5^ The syncytin protein facilitates the binding of cells to form syncytiotrophoblast that provides protection of the embryo from pathogens and, at the same time, enables the transfer of nutrients to the embryo.^5^ Another example is HERV env protein conferring cellular resistance to superinfection by exogenous retroviruses that can potentially be oncogenic or immunosuppressive.^6^

A group of HERVs that has come to the fore in recent years in the study of neurodegenerative diseases is HERV‐K, also known as human mouse mammary tumor virus‐like 2. The letter *K* (the symbol for lysine) was assigned to this group because of its inferred utilization of lysine tRNA primer for initiating reverse transcription.^7^ HERV‐K is thought to be the most active of all HERVs and has been studied mainly in the context of neurodegenerative diseases, in particular amyotrophic lateral sclerosis (ALS)^8^, ^9^, ^10^ and, to a lesser extent, frontotemporal dementia (FTD),^11^ which are considered to exist on the same disease spectrum because of the substantial overlap in phenotypic traits in the two diseases. In both ALS and FTD, HERV‐K levels are elevated when comparing with healthy controls.^11^, ^12^, ^13^ Growing evidence indicates that HERV‐K is important in human health, and that investigating their fundamental properties could facilitate the development of novel therapies for neurodegenerative diseases.

However, very little is known about HERV‐K in PD. In an early study, the level of HERV‐K *pol* mRNA transcript was shown to be extremely low or undetectable in PD brain samples, as measured by quantitative polymerase chain reaction (PCR).^10^ In another study, an ontology enrichment analysis inferred that polymorphic HERV‐K could be linked to PD.^10^ Based on what we know of HERV in human physiology, we hypothesized that HERV‐K is altered in PD and is related to PD progression. The aims of our study were to explore and unravel the relationship between HERV‐K and PD and to examine whether HERV‐K could serve as a potential progression biomarker for PD.

## Subjects and Methods

### Human Brain Tissues

Postmortem brain tissue samples (Table 1) were obtained from Sydney Brain Bank and NSW Brain Tissue Resource Centre. Ethical approvals were acquired from the human research ethics committees of the University of New South Wales (approval no. HC16568) and the University of Sydney (approval no. 2020/707). All brain donors underwent standardized assessments in life and standardized neuropathological examination. Patients with PD (n = 8) met current consensus diagnostic criteria for PD and were without other significant neuropathologies. Controls (n = 10) were without neurological, psychiatric, or neuropathological diagnoses.

**Table 1:** Demographics of postmortem brain tissues from patients with PD and controls

ID no.	Case	Age (y)	Sex	Disease duration (y)	Postmortem interval (h)	Braak stage	Cause of death
1	PD	79	M	17	42	6	Septicemia
2	PD	82	M	22	19	6	Cardiorespiratory failure
3	PD	84	M	12	5	5	Cholangitis
4	PD	79	M	7	17	5	Acute myocardial infarction
5	PD	81	F	22	29	6	Cardiorespiratory failure
6	PD	82	F	8	9	5	Pneumonia
7	PD	73	M	13	20	6	Cardiorespiratory failure
8	PD	82	M	7	22	6	Cardiorespiratory failure
9	Control	94	M	–	24	0	Cardiovascular failure
10	Control	87	F	–	24	0	Acute peritonitis
11	Control	89	M	–	22	0	Cardiovascular failure
12	Control	84	M	–	9	0	Pancreatic cancer
13	Control	60	M	–	25	0	Infection
14	Control	72	F	–	25	0	Cardiac
15	Control	80	M	–	12	0	Respiratory
16	Control	67	M	–	29	0	Cardiac
17	Control	81	F	–	35	0	Cancer
18	Control	75	M	–	34	0	Cardiac

Abbreviations: PD, Parkinson's disease; M, male; F, female.

### Human Plasma

Plasma aliquots from a prior study conducted by our group to investigate glucocerebrosidase activity in patients with PD were used.^14^ PD cases (n = 25) were clinically diagnosed according to established criteria.^15^ Healthy controls (n = 25) were age and sex matched and had no neurological or psychiatric disorders or no first‐degree relatives diagnosed with PD. An exclusion criterion was the current use of nonsteroidal anti‐inflammatory medication. The study was approved by the University of Sydney Human Research Ethics Committee (approval no. 2016/363). Blood samples were obtained following written informed consent from the participant and/or primary carer as legal representative. Venous blood was collected into 8‐mL CPT vacutainers (BD Biosciences, Franklin Lakes, NJ, USA) and centrifuged at 1800*g* for 20 minutes at room temperature (RT), and plasma was collected, aliquoted, and stored at −80°C until use.

### Immunofluorescence

Formalin‐fixed, paraffin‐embedded sections (10 μm) from superior frontal and fusiform cortices were deparaffinized in xylene and rehydrated through graded ethanol. Antigen retrieval was first with 95% formic acid at RT for 5 minutes and then Tris‐EDTA buffer using a pressure cooker (Aptum Bio Retriever 2100; Aptum Biologics Ltd, Southampton, UK) at a peak temperature of 121°C and gradually cooled to RT. Sections were blocked with 2.5% donkey serum and 1% BSA in phosphate‐buffered saline for 1 hour at RT and then incubated with the cocktail of primary antibodies, including ERVK‐7 polyclonal antibody^16^ (PA5‐49515, rabbit, 1:100; Invitrogen, Melbourne, Australia) and glial fibrillary acidic protein (GFAP; a85307, chicken, 1:400; Abcam, Melbourne, Australia) at 4°C for two nights. Sections were washed and incubated in the corresponding Alexa Fluor 488/568/647 secondary antibodies (1:250) and 4′,6‐diamidino‐2‐phenylindole (DAPI; 1 mg/mL; Cat. No. D9542; Sigma, St. Louis, MO, USA) for 2 hours at RT. Next, the slides were treated with 70% Sudan Black for 30 minutes and 10 mM CuSO_4_ in 50 mM ammonium acetate buffer (pH 5.0) for 1 hour to quench autofluorescence before coverslipping with anti‐fade fluorescence mounting medium (S3023; DAKO, Santa Clara, CA, USA) and then sealed with nail polish. Negative controls (without primary or secondary antibodies) were performed for each immunohistochemistry run, and no fluorescence signals were detected in each case.

### Immunohistochemistry

Formalin‐fixed, paraffin‐embedded sections (10 μm) from superior frontal and fusiform cortices were deparaffinized in xylene and rehydrated through graded ethanol, followed by antigen retrieval with Tris‐EDTA buffer using a pressure cooker (Aptum Bio Retriever 2100; Aptum Biologics Ltd) at a peak temperature of 121°C and gradually cooled to RT. Endogenous peroxidase was blocked with 1% hydrogen peroxide in 50% ethanol. Sections were blocked with 5% normal horse serum, then incubated with ERVK‐7 polyclonal antibody^16^ (PA5‐49515, rabbit, 1:100; Invitrogen) at 4°C for two nights, followed by the secondary antibody ImmPRESS‐AP Anti‐Rabbit IgG Polymer Detection Kit (MP‐5401; Vector Laboratories, Newark, CA, USA) as per the manufacturer's instructions. Sections were then counterstained with hematoxylin and coverslipped. Negative controls (without primary antibody or secondary antibody) were performed for each immunohistochemistry run, and no phosphatase signals were detected.

### Microscopy Imaging and Quantification

For immunohistochemistry, stained sections were scanned using an Olympus VS120 Slide Scanner with the same focus and exposure settings. For immunofluorescence, multiple sections were examined, and representative images were captured with a Nikon C2 confocal microscope and associated Nikon NIS Elements software (version 4.60). Images were adjusted for contrast and converted to TIFF format on Fiji software (ImageJ version 2.0.0‐rc‐69/1.52p). HERV‐K env–positive astrocytes were quantified using QuPath software (v0.3.2).

### Protein Extraction

Tris‐buffered saline (TBS) and SDS‐soluble proteins were serially extracted from 100 mg of fresh‐frozen brain tissues, as previously described.^17^ In brief, tissues were homogenized in 10 vol of TBS homogenization buffer (20 mM Tris, 150 mM NaCl [pH 7.4], 5 mM EDTA, 0.02% sodium azide) containing protease inhibitor cocktail (Roche) using Qiagen TissueLyser (3 × 30‐second, 30‐Hz cycles), followed by centrifugation at 100,000*g* for 1 hour at 4°C, with supernatant collected as TBS‐soluble fraction. The pellet was resuspended in SDS solubilization buffer (TBS homogenization buffer containing 5% SDS) using 3 × 30‐second, 30‐Hz cycles with TissueLyser and centrifuged at 100,000*g* for 30 minutes at 25°C, with supernatant collected as SDS‐soluble fraction. Protein concentration was measured using a bicinchoninic acid assay (Pierce BCA Protein Assay Kit) following the manufacturer's instructions.

### Western Blotting

Protein lysates (10 μg) were heated with sample buffer (3.2% SDS, 32% glycerol, 0.16% bromophenol blue, 100 mM Tris–HCl [pH 6.8], 8% 2‐mercaptoethanol). They were then electrophoresed on Criterion Stain‐free 4%–20% SDS‐PAGE gels (Bio‐Rad, Sydney, Australia) and transferred onto nitrocellulose membranes at 100 V for 30 minutes. The membranes were blocked with TBS containing 5% nonfat dry milk and probed with ERVK‐7 polyclonal antibody (1:1000, PA5‐49515; Invitrogen) or GFAP polyclonal antibody (1:10,000, GA52461‐2; Agilent) overnight at 4°C. They were then washed three times in TBS containing 0.1% Tween 20 and incubated with horseradish peroxidase–conjugated secondary antibody for 2 hours at RT. Signals were detected using enhanced chemiluminescence and Gel Doc System (Bio‐Rad). The blots were stripped and probed for housekeeper proteins β‐actin or GAPDH. The signal intensity was quantified using Image Lab (Bio‐Rad) and National Institutes of Health (NIH) ImageJ software (v1.45s).

### Nucleic Acid Extraction

Total nucleic acids were extracted from plasma (300 μL) using QIAamp Viral RNA Mini Kit (Qiagen) following the manufacturer's instructions. Plasma samples were first centrifuged for 10 minutes to remove any cellular debris. Extracted nucleic acids were not treated with DNase because HERV‐K viral particles contain both RNA and DNA.^18^, ^19^, ^20^ Genomic DNA (gDNA) was extracted from whole blood using the QIAamp Blood Mini Kit (Qiagen) following the manufacturer's instructions.

### HERV‐K Digital Droplet Polymerase Chain Reaction Assay

HERV‐K *env* load and copy number were measured using digital droplet PCR (ddPCR) as previously described.^21^ A total of 2.5 μL of extracted nucleic acids was added to a reaction mixture containing 12.5 μL of ddPCR Supermix (no dUTP) (Bio‐Rad), 1.25 μL of HERV‐K *env* primer (900 nm) and probe (250 nm) mix, 1.25 μL of ddPCR RPP30 copy number assay, and 7.5 μL of nuclease‐free water. Droplets were generated on QX100 Droplet Generator (Bio‐Rad), and PCR was run on C1000 Thermal Cycler (Bio‐Rad). The PCR program was 10 minutes at 95°C, 40 cycles of 30 seconds at 95°C and 1 minute at 60°C, and 10 minutes at 95°C. Droplets were assessed in a QX200 Droplet Digital PCR System (Bio‐Rad). HERV‐K levels were recorded as the ratio of HERV DNA copies to RPP30 DNA copies. Primer and probe sequences were as follows: HERV‐K forward: 5′‐ATTTGGTGCCAGGAACTGAG‐3′; HERV‐K reverse: 5′‐GCTGTCTCTTCGGAGCTGTT‐3′; and HERV‐K Probe: 5′‐6‐FAM‐AGGAGTTGCTGATGGCCTCG‐Iowa Black FQ‐3′ (Bio‐Rad). RPP30 copy number assay Hex labeled (dHsaCP2500350; Bio‐Rad) was used.

### GFAP Assay

The ultrasensitive single‐molecule array Human Neurology 4‐Plex A assay (Quanterix; Cat. No. 102153) and Quanterix HD‐1 Analyzer (Quanterix) were used for the measurement of GFAP. In brief, sample aliquots were sent to GeneWorks (Melbourne, Australia) on dry ice with the Quanterix assay performed on a fee‐for‐service basis following the manufacturer's instructions and a 1:4 dilution of sample. A seven‐point calibration curve and quality controls for each analyte were included. The calibration curve range was 0–9000 pg/mL. Concentrations of GFAP in samples and quality controls were interpolated from the calibration curve using a cubic curve fit (1/*y* ^2^ weighted). Duplicates were used for intra‐assay and interassay precision testing. A coefficient of variation (CV) >12% cutoff was chosen based on the assay datasheet, which reported a maximum within‐assay CV of 11.3%, which is regarded as good precision.^22^

### Statistical Analysis

Statistical analyses were performed using SPSS Statistics software 26 (IBM, Chicago, IL, USA). For comparisons between PD and control groups, univariate analysis (general linear model), with age and sex as covariates, was used and significance set at *P* < 0.05. Association analyses were performed using Pearson's correlation and statistical significance set at *P* < 0.05. Receiver operating characteristic (ROC) analysis was used to determine the best cutoff for HERV‐K and curves generated in SPSS 26. All other graphs were generated using GraphPad Prism 7.

## Results

### HERV‐K Env Is Colocalized with GFAP in Astrocytes

**FIG. 1:**
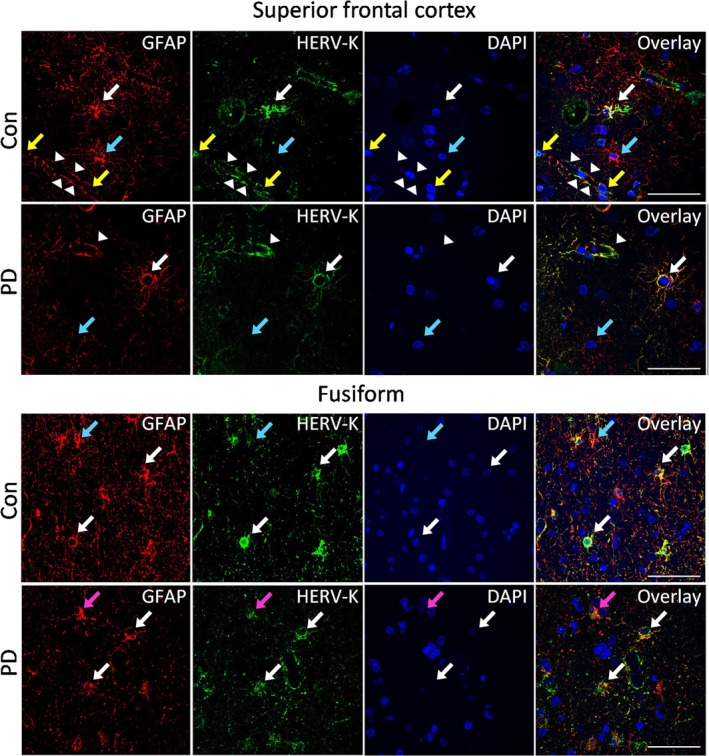
Analysis of human endogenous retrovirus K (HERV‐K) env localization in Parkinson's disease (PD) and control (Con) brain tissues. HERV‐K env is predominantly colocalized with glial fibrillary acidic protein (GFAP) in astrocytes in the superior frontal and fusiform cortices. HERV‐K env is expressed in reactive astrocytes (white arrows) and quiescent astrocytes in PD (white arrows). There were also reactive astrocytes without HERV‐K env (cyan arrows). In terms of astrocyte cellular localization, HERV‐K env is present in the cytoplasm (white arrows), endfeet (white arrowheads), and occasionally the nucleus (magenta arrows). HERV‐K env–expressing blood cells were observed in controls (yellow arrows). Scale bars, 50 μm. [Color figure can be viewed at wileyonlinelibrary.com]

We analyzed in situ HERV‐K env expression in the superior frontal and fusiform cortices of PD and control brain using immunofluorescence and confocal microscopy. Tissues from other affected regions, such as the substantia nigra, were not available from the brain banks and therefore could not be analyzed. We found that HERV‐K env was extensively expressed in both cortices in PD and control brain (Fig. 1). HERV‐K env was predominantly colocalized with GFAP in astrocytes in both brain regions (Fig. 1). In controls, HERV‐K env was expressed in some, but not all, reactive astrocytes, whereas in PD, it was expressed in quiescent astrocytes (Fig. 1). At the subcellular level, it was localized to the astrocytic cytoplasm, endfeet, and nucleus (Fig. 1).

### HERV‐K–Positive Cells Are Decreased in PD Brain

**FIG. 2:**
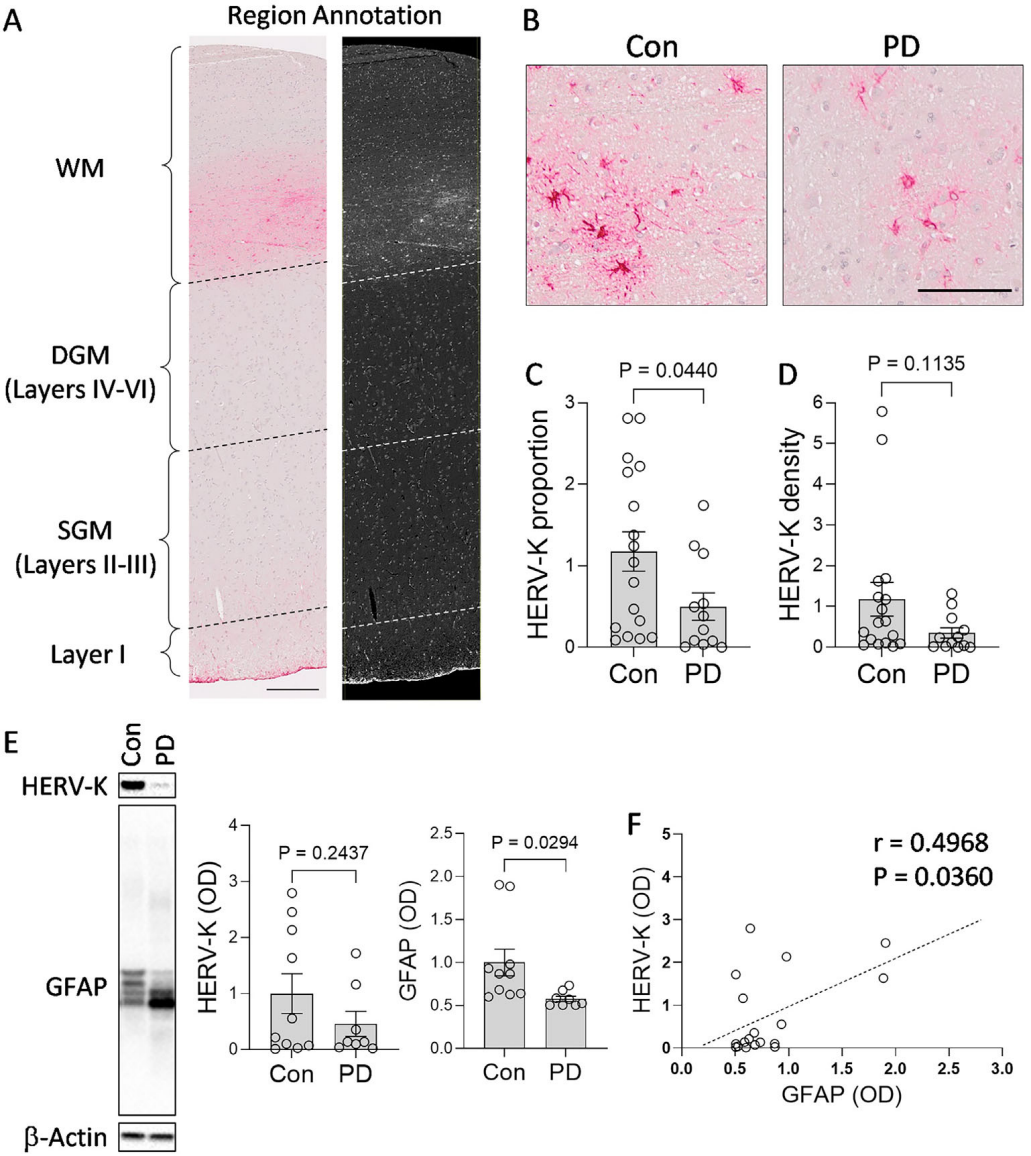
Quantitative measurement of human endogenous retrovirus K (HERV‐K) env in brain tissues. (**A**) Region annotation of cortices immunostained. White matter (WM), deep gray matter (DGM), superficial gray matter (SGM). Scale bar, 250 μm. (**B**) HERV‐K protein (pink) is predominantly expressed in astrocytes. Scale bar, 100 μm. (**C**) Quantitative measurement of proportion of HERV‐K env–positive cells (HERV‐K proportion). (**D**) Quantitative measurement of density of HERV‐K env–positive cells (HERV‐K density). (**E**) Measurement of HERV‐K env and glial fibrillary acidic protein (GFAP) in patients with Parkinson's disease (PD) and controls (Con) by Western blotting. (**F**) Analysis of correlation between HERV‐K env and GFAP. [Color figure can be viewed at wileyonlinelibrary.com]

To determine whether HERV‐K env expression was altered in PD astrocytes, we immunostained tissues from the superior frontal and fusiform cortices with HERV‐K env antibody and quantitated the signal using QuPath software. The white matter layer, where the signal was most prominent (Fig. 2A), was quantitated. Once again, in controls, HERV‐K env was expressed predominantly in reactive astrocytes, whereas in PD, it was expressed predominantly in quiescent astrocytes (Fig. 2B). The proportion of HERV‐K env–positive cells was significantly decreased in PD cortices compared with controls (Fig. 2C). Likewise, the density of the signal trended lower in PD compared with controls (Fig. 2D). We also assessed HERV‐K env and GFAP levels by Western blotting and found that HERV‐K env trended lower in PD compared with controls, whereas GFAP was significantly decreased in PD compared with controls (Fig. 2E). There was also a significant association between HERV‐K env and GFAP (Fig. 2F).

### HERV‐K Load Is Decreased in PD Blood

**FIG. 3:**
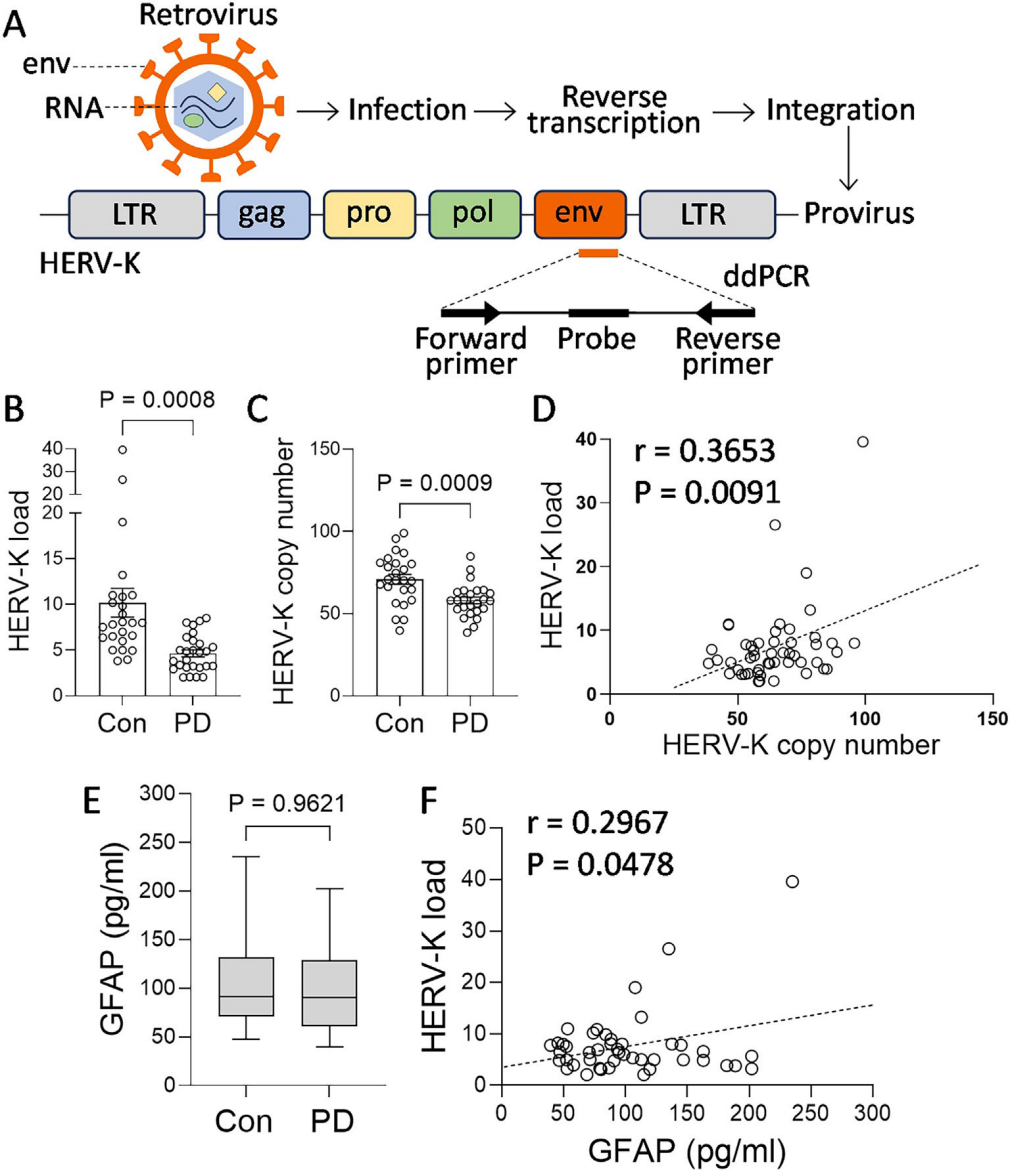
Assessment of human endogenous retrovirus K (HERV‐K) load in Parkinson's disease (PD) blood. (**A**) Genomic structure of HERV‐K and measurement of HERV‐K *env* by digital droplet polymerase chain reaction (ddPCR). (**B**) Analysis of HERV‐K load in PD plasma compared with controls (Con). (**C**) Analysis of HERV‐K copy number in the gDNA of peripheral blood mononuclear cells. (**D**) Analysis of correlation between HERV‐K load and HERV‐K copy number. (**E**) Measurement of glial fibrillary acidic protein (GFAP) in PD and control plasma. (**F**) Analysis of correlation between HERV‐K load and GFAP in plasma. [Color figure can be viewed at wileyonlinelibrary.com]

Because HERV‐K env is associated with GFAP in the brain, we were interested in whether there was also an association in the blood that would support the idea of considering these peripheral markers as representing similar changes in PD brain. Plasma and buffy coats were prepared from sporadic PD (n = 25) and control (n = 25) blood with the buffy coats further processed to extract gDNA from peripheral blood mononuclear cells (PBMCs). A set of specific primers was designed to amplify the HERV‐K *env* gene by ddPCR (Fig. 3A). We found that HERV‐K *env* load in the plasma was significantly decreased in PD compared with controls (Fig. 3B). We also measured HERV‐K *env* copy number in the PBMC gDNA and found that it was also significantly decreased in PD compared with controls (Fig. 3C). There was a strong correlation between HERV‐K *env* load and HERV‐K *env* copy number (Fig. 3D). Finally, we measured GFAP levels in PD and control plasma (Fig. 3E), and they were positively correlated to HERV‐K *env* load (Fig. 3F), consistent with the interaction observed in the brain. These results show that HERV‐K load is decreased in PD plasma as a result of decreases in the genomic copy number of HERV‐K, and that the loss of HERV‐K is related to astrocytic brain changes in PD.

### Loss of HERV‐K in Blood Is Related to PD Progression

**FIG. 4:**
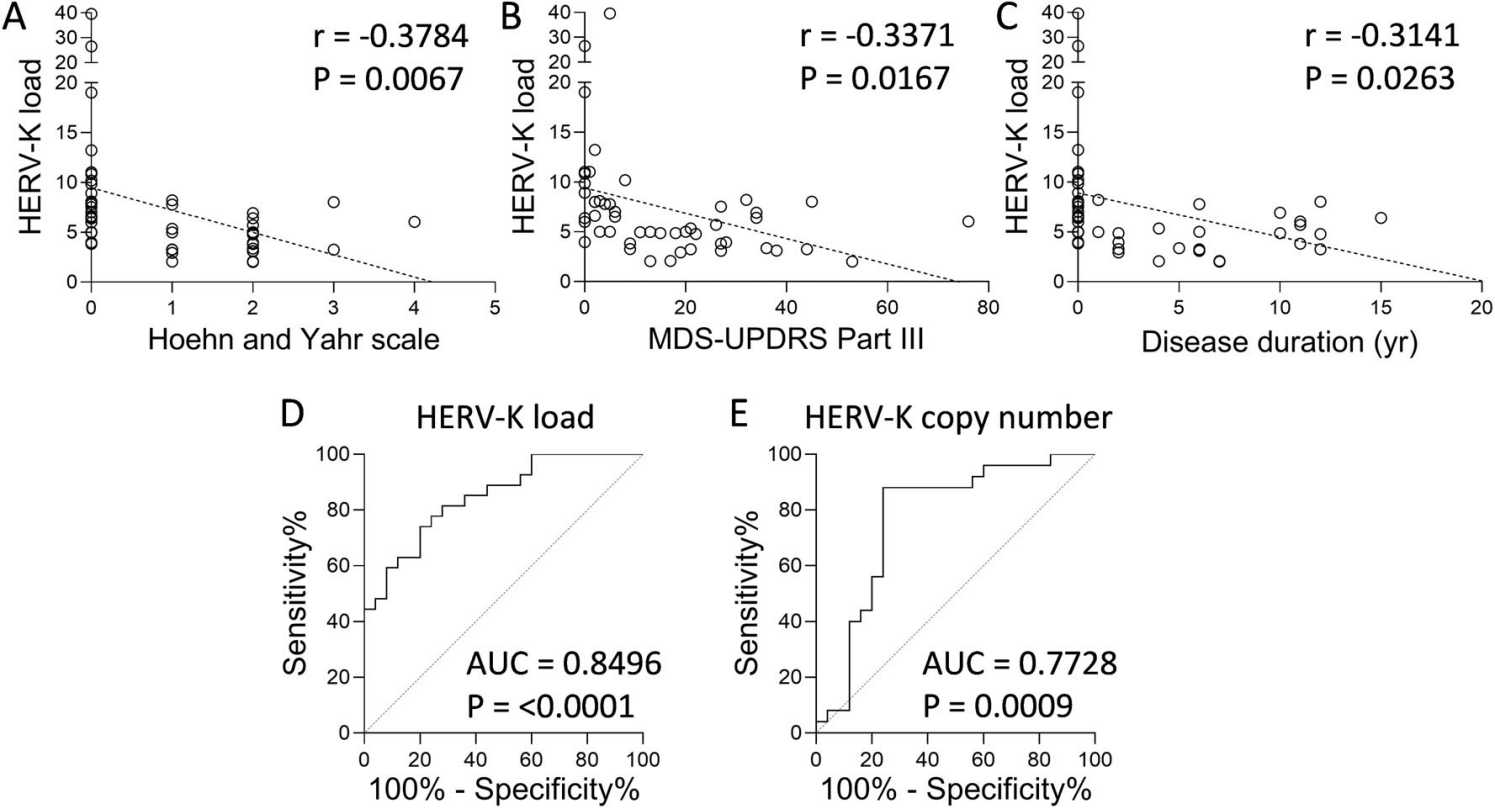
Human endogenous retrovirus K (HERV‐K) load is related to Parkinson's disease (PD) progression. (**A**) Analysis of correlation between HERV‐K load and Hoehn and Yahr Scale. (**B**) Analysis of correlation between HERV‐K load and Movement Disorder Society Unified Parkinson's Disease Rating Scale (MDS‐UPDRS) Part III. (**C**) Analysis of correlation between HERV‐K load and disease duration. (**D**) Receiver operating characteristic (ROC) curve for HERV‐K load. (**E**) ROC curve for HERV‐K copy number. AUC, area under the curve.

We then analyzed HERV‐K load in relation to two PD clinical rating scales, the Hoehn and Yahr Scale and the Movement Disorder Society Unified Parkinson's Disease Rating Scale (MDS‐UPDRS) Part III, both of which evaluate longitudinal motor function in patients with PD. We found that HERV‐K load was inversely correlated to both scales (Fig. 4A,B). In addition, we analyzed HERV‐K load in terms of disease duration and found that it was also inversely correlated to disease duration (Fig. 4C). These results suggest that loss of HERV‐K is related to PD progression. To explore whether the loss of HERV‐K could contribute as a potential diagnostic biomarker for PD, we then generated ROC curves for HERV‐K load and HERV‐K copy number and found that both yielded significant areas under the curve (AUCs) (Fig. 4D,E).

## Discussion

HERVs are proviral retrotransposons that constitute an estimated 8% of the human genome,^23^ and their importance in human physiology is increasingly being recognized. Multiple lines of evidence indicate that HERVs participate in protective measures in a number of cellular processes, including safeguarding placenta morphogenesis, increasing antioncogenic capability, and conferring resistance to viral infection. In recent years, the importance of understanding the facet of HERV‐K in neurodegenerative diseases is increasingly recognized, with the potential of developing novel therapeutic strategies for neurodegenerative diseases. However, very little is known about HERV‐K in PD, with only two papers reporting on the subject.^10^, ^24^ To address this knowledge gap, we undertook a series of studies investigating the relationship between HERV‐K and PD. We showed that HERV‐K protein is predominantly colocalized with GFAP in astrocytes, with decreased expression in PD brain compared with controls. A decreased expression of GFAP in PD brain has been previously shown to correlate with the increased expression of pathological α‐synuclein protein,^25^ and our data show that this may also impact HERV‐K protein levels. Consistent with this, HERV‐K levels were decreased in PD blood compared with controls and were correlated to blood GFAP levels. Furthermore, HERV‐K levels were inversely correlated to PD progression as measured by the Hoehn and Yahr Scale and MDS‐UPDRS Part III. When put together, these results suggest that loss of HERV‐K and GFAP is related to PD progression, and that the expression of both HERV‐K and GFAP could be viewed, possibly, as neuroprotective.

We observed that HERV‐K protein is predominantly expressed in astrocytes in both controls and patients with PD. Very little is known about HERV‐K in the context of astrocytes. In one study, mice that received cells that were transfected with the HERV‐K *env* gene displayed increases in astrocytic GFAP immunoreactivity and improved neuroprotection.^26^ Consistent with this, we found that decreases in HERV‐K protein correlated with decreases in astrocytic GFAP in PD, and that in peripheral blood the loss of HERV‐K and GFAP was associated with PD severity and duration. These data support the idea that astrocytic HERV‐K could be neuroprotective and has been altered in PD. Although PD has traditionally been considered a neuronal autonomous disease, with the deposition of α‐synuclein in neurons as the main pathology, growing evidence indicates that changes to astrocytes contribute to PD vulnerability and pathology.^27^ This is not surprising in that astrocytes support the neurons in a wide variety of processes, including synaptogenesis, neurogenesis, synaptic maintenance, neurotransmitter homeostasis, and energy supply,^28^ and that astrocytes can contribute to initiating neurodegeneration.^29^ We observed that HERV‐K is expressed mainly in reactive astrocytes in controls and more quiescent astrocytes, at reduced levels, in PD. These results suggest that the activity/function of HERV‐K in astrocytes is altered in PD, resulting in a reduced astrocytic neuroprotection and possibly accentuating neurodegeneration. Interestingly, HERV‐K protein was expressed in astrocyte endfeet, which cover approximately 95% of the brain capillary surface, and therefore it is plausible to think that the activity of HERV‐K could also be related to neurovascular functions as well.

The loss of astrocytic HERV has not been reported previously with PD progression. However, a few studies have shown that a group of retrotransposons that belong to the SINE‐VNTR‐Alu (SVA) class is positively associated with PD progression, as well as dopaminergic degeneration.^30^ Likewise, SVAs are positively associated with PD severity as measured by UPDRS Part II score, UPDRS total score, and modified Schwab and England ADL (activities of daily living) scale.^31^ Furthermore, members of another class of retrotransposons, long interspersed nuclear elements (LINEs), are positively associated with longitudinal changes in PD progression markers.^32^ Both SVAs and LINEs are, however, structurally very different from HERVs, and their increase compared with HERV decrease supports functional differences over the disease course.

Expression of HERV‐K is universal throughout the human body, irrespective of disease, with the brain being the highest, as well as expressing a broad range of HERV‐K members.^3^ It is thought that HERV‐K was derived from an independent infection by an exogenous retrovirus,^33^ and that the mechanism of its propagation appears to be either reinfection or replication of an existing provirus.^34^ It is thought that there are multiple copies of HERV‐K located throughout the genome on at least 12 of the chromosomes.^21^ We found that HERV‐K levels are overall decreased in PD compared with controls, which is in contrast with the observations made in ALS and FTD. In ALS serum and CSF, HERV‐K DNA, protein, and activity are all elevated compared with healthy controls.^12^, ^13^ In FTD serum and brain tissues, HERV‐K DNA and protein are elevated compared with controls.^11^ In both ALS and FTD, however, increases in the neuropathogenic protein TDP‐43, a transcriptional factor, are thought to promote HERV‐K transcription by binding to the TAR element within the HERV‐K long terminal repeats (LTR).^9^, ^11^ TDP‐43 is not normally elevated in PD brain or pathologically associated with PD; therefore, it is likely that the mechanism involved in altering the HERV‐K level in PD is quite different from that of ALS and FTD.

In our study, we found that both HERV‐K load and copy number in blood were good discriminators of PD from controls, with reduced HERV‐K load providing a greater AUC value and significance for differentiating patients with PD. HERV‐K load was also shown to be positively associated with blood GFAP, which has previously been considered as a biomarker for PD.^35^ Our data suggest that the loss of HERV‐K may be a good progression biomarker for PD, although HERV‐K has also been considered as a potential biomarker for aging itself, as well as a potential therapeutic target for alleviating tissue and cellular senescence.^36^ This would be consistent with a neuroprotective function. It would be interesting to see whether HERV‐K expression impacts on some of the promising prognostic biomarkers for PD onset and progression.^37^, ^38^ In fact, HERV‐K, in combination with other promising prognostic markers, such as DOPA decarboxylase,^38^ could be used to develop a diagnostic/prognostic multiplex biomarker assay for PD.

In conclusion, we demonstrated that loss of HERV‐K is related to PD progression, and that HERV‐K is likely to be neuroprotective. Although further work is needed to verify our findings in larger cohorts, our study has provided new insights into an unrecognized pathway in PD and opened a new area of research for better understanding PD and developing novel therapeutic strategies for PD. With improvements in single‐cell RNA sequencing and spatial transcriptomics, there is a greater scope for determining the extent of HERV‐K presence in the human genome and for better understanding of its role in human physiology.

## Author Roles

W.S.K. conceived, developed, and supervised the project; analyzed the data; and wrote the manuscript. Y.F. carried out immunohistochemistry and revised the manuscript. G.L.A. and K.P. carried out ddPCR and Western blotting. P.Y. carried out biochemical assays. G.M.H. provided laboratory and salary funding, provided expert advice, and revised the manuscript. N.D. acquired the blood samples, provided expert advice, and revised the manuscript. All authors reviewed and approved the manuscript.

## Financial Disclosures

The authors declare no competing interests.

## Data Availability

On reasonable request, data are available upon relevant ethical approval by contacting the corresponding author.
